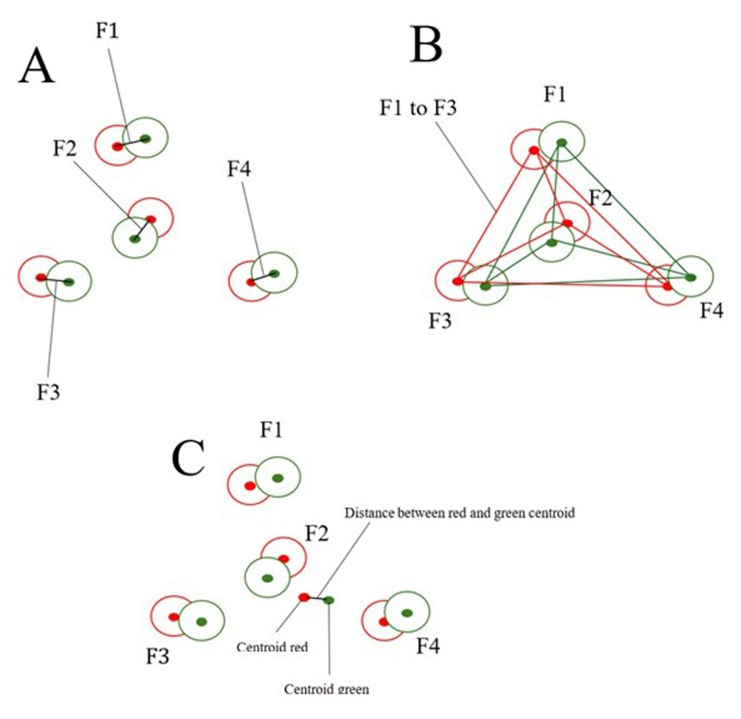# Feasibility of Mixed Reality-assisted physician-modified endografts

**DOI:** 10.1016/j.jvscit.2025.101889

**Published:** 2025-06-21

**Authors:** Johannes Hatzl, Christian Uhl, Jana Ebner, Alexandra Marquardt, Dimitrios David Papazoglou, Nina Bauer, Katrin Meisenbacher, Daniel Henning, Andreas Sebastian Peters, Dittmar Böckler

**Affiliations:** a Department of Vascular and Endovascular Surgery, University Hospital Heidelberg, Heidelberg, Germany; b Department of Vascular Surgery, RWTH Aachen, Aachen, Germany; c Brainlab AG, Munich, Germany; d Department of Vascular Surgery, Inselspital, University Hospital Bern, University of Bern, Bern, Switzerland

**Keywords:** Augmented reality, Complex aortic aneurysm repair, Endovascular repair, Extended reality, Fenestrated repair, Mixed-reality, Physician-modified, PMEG, PMSG

## Abstract

This study aimed to develop and evaluate a standardized workflow for physician-modified endografts using Mixed Reality (MxR) technology, and to compare it with a conventional method in a phantom model experiment. The experiment consisted of two parts. In the first part, the feasibility of using a virtual MxR overlay to guide fenestration marking on a phantom stent graft was tested. Thirty-two observers each marked four fenestrations (F1-F4), totaling 128 markings. In the second part, 12 observers performed both the MxR-assisted workflow and a conventional method. Outcomes included positional accuracy, procedure time, usability, and number of required reattempts. Accuracy was assessed by comparing absolute and relative distances from ideal positions and fenestration centroids. The required time of the workflow was recorded. Usability was evaluated using the System Usability Scale (SUS) and the Post-Study System Usability Questionnaire (PSSUQ). In the feasibility test, mean deviations from ideal positions were 1.3 mm (F1), 1.0 mm (F2), 1.0 mm (F3), and 1.5 mm (F4). Centroid errors were 0.7 mm (clock position) and 0.5 mm (cranio-caudal). The workflow took 5:31 minutes on average, with step 2 requiring 2:55 minutes. SUS and PSSUQ scores indicated high usability (84.2/100 and 1.8/7, respectively). In the comparative analysis, the MxR group showed comparable accuracy to the conventional method but required fewer reattempts (3 vs 10) and less time (6.7 vs 14.6 minutes; *P* < .01). Usability ratings were significantly higher for MxR (SUS, 85.1 vs 43.2; *P* < .01). The MxR-assisted workflow enabled accurate, efficient, and user-friendly physician-modified endografts planning. At least in this experimental setup, it outperformed the conventional method in usability and speed, supporting its potential for broader clinical applications.

Physician-modified endografts (PMEGs) have emerged as an effective endovascular treatment option for various aortic pathologies.^1^ It is especially useful in emergent settings where manufacturing of custom-made devices (CMDs) is not feasible, or in cost-sensitive health care systems.^2^ Nevertheless, PMEG creation is associated with significant stent graft manipulation outside the instructions for use and could be improved regarding methodological standardization. After manual measurements of fenestration positions, the conventional methods include marking and creation of target vessel fenestrations on the stent graft using some form of physical reference on the stent graft for orientation, such as drawing reference lines or punch cards.^3^^,^^4^ Alternatively, three-dimensional (3D)-printed aortic models have been used for PMEG creation with high precision and technical success.^5^^,^^6^ However, the production and sterilization time is a major handicap for this technique in emergent settings. Thus, the available methods for PMEG manufacturing have potential for improvement, increasing the ease of use, accuracy of fenestration placement, and minimizing the time required.

Mixed Reality (MxR) is a digital imaging technology that projects virtual objects in the real-world and enables interaction with such objects by using a head-mounted display (HMD). The first MxR-assisted PMEG has been reported as a case report in 2022.^7^ Our aim was to develop a standardized workflow of MxR-assisted PMEG manufacturing and investigate its feasibility as well as compare it with a conventional control.

## Materials and methods

### Overview of the MxR-assisted modification process

The MxR-assisted modification process involves five steps.

**Fig 1:**
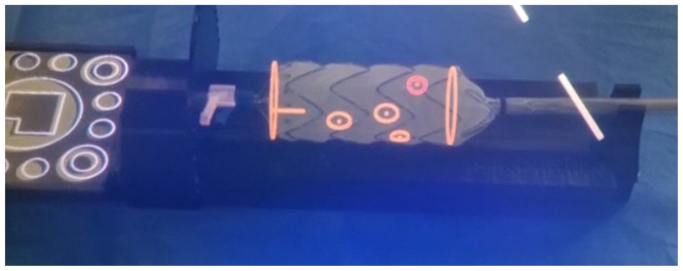
Stent graft (Valiant Captivia, Medtronic plc), registered with the virtual object, positioned in the template. Point of view of the surgeon performing physician-modified endograft (PMEG) planning.
(3) Registration: The stent graft is then semideployed or deployed and positioned in the PMEG template. The template is a custom design by our group and enables the stent graft to be accurately placed in a defined position related to a MxR marker, which was provided by Brainlab AG. The template also allows rotation and tracking of the stent graft’s longitudinal rotational position. Due to the known dimensions of the template, the virtual object can be registered with the stent graft using the MxR marker, which has a known dimensional relation with the template and therefore with the physical stent graft position.
(4) Rotation: Following registration, the stent graft is rotated along its longitudinal axis to ensure that stent struts are avoided. While the stent graft is rotated, the virtual object remains fixed. Therefore, the user can identify the most advantageous stent graft position, in which all fenestrations are unaffected by stent struts. In theory, the stent graft can be rotated 360°. Rotation to identify the most advantageous position avoiding struts is demonstrated in the Supplementary Video (online only) with a Valiant Captivia stent graft (Medtronic).
(5) Marking and modification: Finally, using a sterile pen, the positions of the fenestrations are marked. For marking purposes, a physical dial displaying the current clock position of the stent graft is attached to the template. This ensures that the fenestration that is marked next can be rotated directly in the field of view of the observer. The dial design was added for the second part of the experiment. Subsequently, the stent graft can be modified according to routine practice. The marking of the stent graft is illustrated in Fig 2. All the required materials are displayed in Supplementary Fig 1 (online only).

(1) Aortic morphology assessment: The surgeon assesses aortic morphology using standard software, such as 3mensio (Pie Medical Imaging BV), to determine the baseline characteristics of the stent graft. This includes length, diameter, and the number and size of required fenestrations. For each fenestration, the height, width, distance from the proximal stent graft edge, and clock positions are recorded. For the purposes of the experiments in the present study, a hypothetical stent graft was designed with a total length of 49 mm and a diameter of 32 mm. The stent graft was designed with four fenestrations for the coeliac trunk (F1) and the superior mesenteric artery (F2), as well as the right (F3) and left (F4) renal arteries. F1 and F2 measured 8 mm in height and width, F1 was positioned 15 mm below the proximal edge at 12:30, F2 was positioned 31 mm below the proximal edge at 12:00, F3 and F4, each with 6 mm in height and width, were positioned at 38 mm and 42 mm, and at the 10:15 and 3:00 clock position, respectively.
(2) Virtual object: A virtual stent graft model is generated based on the desired specifications from step 1 using a software prototype (Brainlab AG). The virtual object generation process consists of two main steps: (1) stent graft configuration and (2) transfer to the MxR environment. Stent graft configuration means to input the stent graft and fenestration dimensions as well as fenestration positions according to step 1 into the software, using a graphical user interface. Subsequently, by visually scanning a software-generated QR code on the monitor while wearing the HMD, the virtual object is transferred into the MxR environment and projected into the user’s field of view. The perspective of the observer performing PMEG manufacturing is displayed in Fig 1.

**Fig 2:**
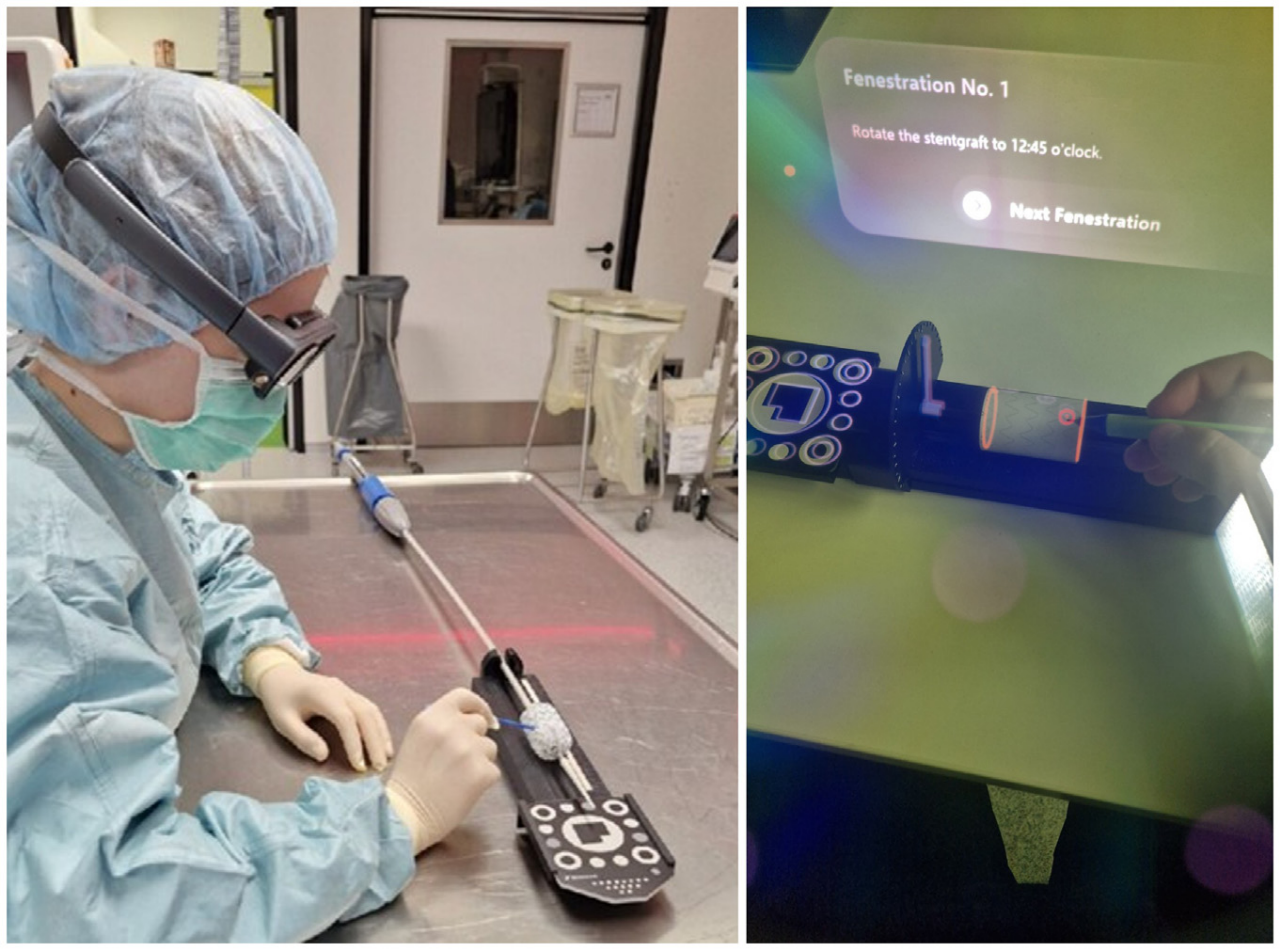
Template and phantom stent graft, registered with the virtual object, positioned in the template. Point of view of the observing surgeon performing part I of this experiment.

### Part I of the experiment: feasibility of fenestration positioning based on a MxR virtual object overlay

**Fig 3:**
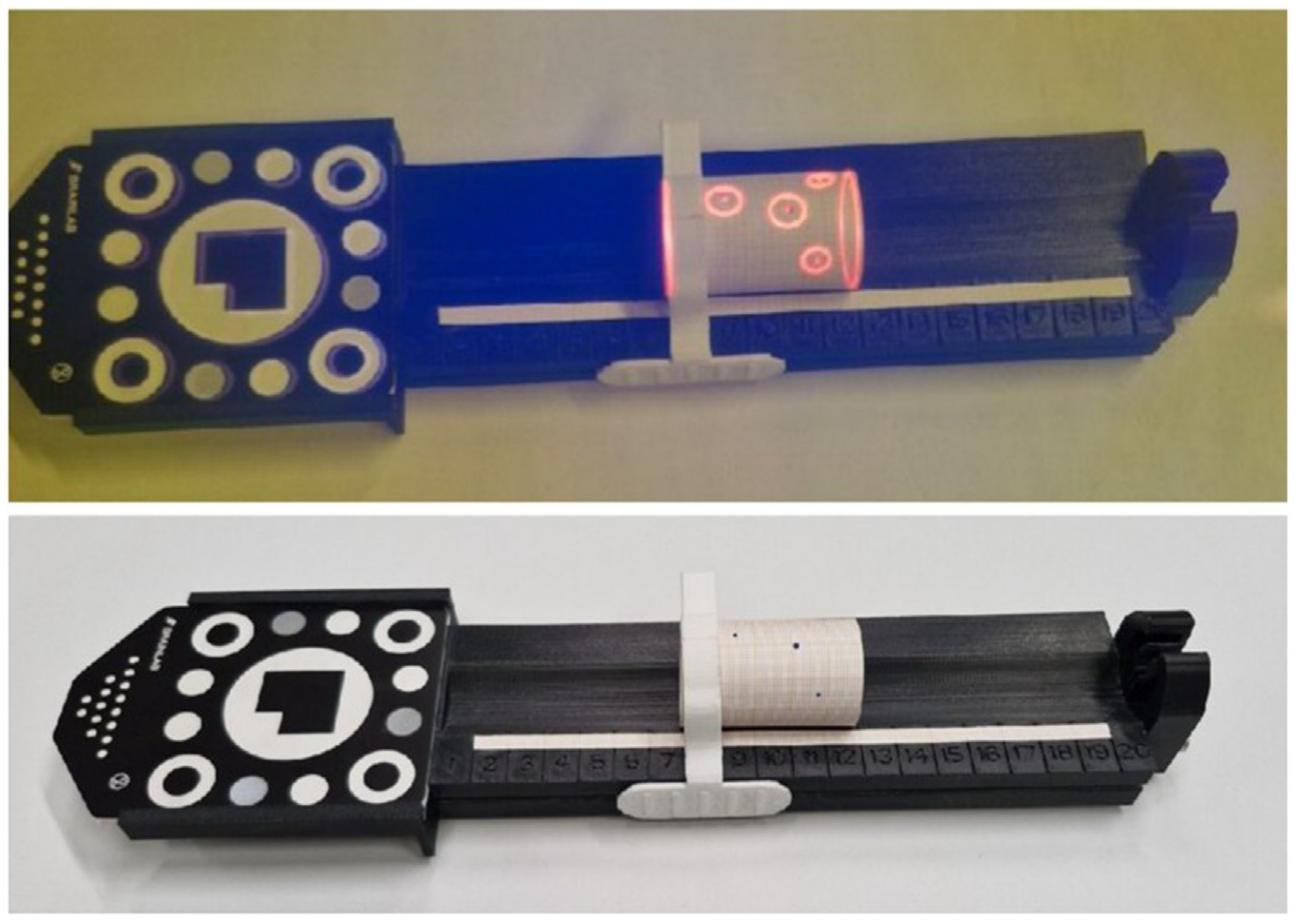
Surgeon performing back table modification of stent graft using the Mixed Reality (MxR)-assisted workflow (illustrative purposes, the experiment was conducted in a nonsterile environment; on display is an Endurant II Cuff (Medtronic plc).

For the first part of the experiment, 32 observers performed the MxR-assisted PMEG workflow (steps 2-5). All four steps were initially demonstrated to each observer individually. Following this demonstration, each observer performed the workflow’s steps 2 to 5. The workflow ended when the marking of the fenestrations was completed. Observers had no or minimal experience with PMEG. Eight of the 32 observers were trained physicians with varying experience in endovascular aortic repair (25%). For the purposes of this experiment, a phantom stent graft was constructed. It was 32 mm in diameter and 49 mm in length measuring 3D-printed tube (Fig 3). The tube was wrapped with paper with a millimeter scale. Thereby, exact measurements of positional errors of the performed markings and a comparison with the ideal positions according to the intended stent graft design (step 1) were performed. Stent struts were omitted for this part of the experiment. Following the workflow, all observers were tasked with the System Usability Scale (SUS) as well as the Post-Study System Usability Questionnaire (PSSUQ), and the millimeter-scaled paper was replaced with a blank one for the next observer.

### Part II of the experiment: comparing the MxR-assisted workflow with a conventional method

**Supplementary Fig 2 (online only):**
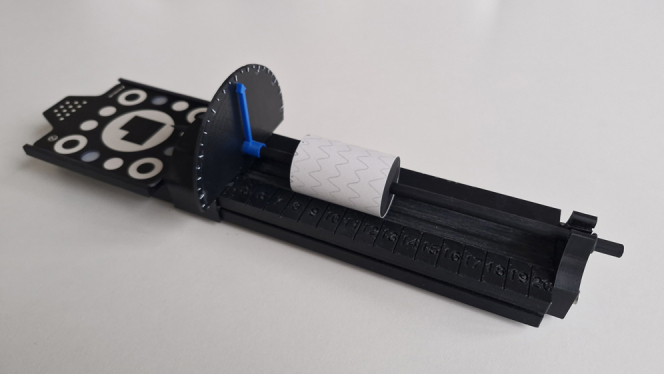


In the second part of the experiment, 12 observers performed the MxR-assisted PMEG workflow (steps 2-5) as described in part I, as well as a conventional method as a control. The conventional method comprised marking the 12-o’clock position with a straight longitudinal line. Afterwards, by trial-and-error principle, a configuration of the prespecified fenestration positions is identified, with the aim of minimizing overlap with stent struts. The phantom stent graft that was used for part II of the experiment is demonstrated in Supplementary Fig 2 (online only). It featured a realistic stent strut configuration. The endpoints of the comparison were positional accuracy, duration, and usability. Additionally, the number of repeated attempts to identify the fenestration configuration with the least overlap with any stent struts was recorded.

### Study endpoints

#### Positional accuracy

Positional accuracy of the fenestration markings was assessed on millimeter-scale paper wrapped around the 3D-printed phantom stent graft models. Measurement of accuracy methodology is displayed in Supplementary Fig 3, *A-C* (online only). The distance of the center of each fenestration marking to the center of the ideal position of the fenestration was measured in millimeters (Supplementary Fig 3, *A*, online only). Additionally, the distances between all centers of fenestration markings on the stent graft models were compared with the distances between all centers of fenestration markings in an ideal configuration (Supplementary Fig 3, *B*, online only). Furthermore, the centroid position of the four fenestrations on the models was compared with the ideal centroid position (Supplementary Fig 3, *C*, online only).

#### Duration

The duration of the MxR-assisted workflow (steps 2-5) was measured. Additionally, the time required to perform step 2 was measured separately.

#### Usability

Usability of the workflow was assessed by each observer using validated tools. To measure the MxR aspect’s usability, the SUS was used. The SUS is expressed as a score ranging from 1 to 100, and 100 indicates excellent usability. It consists of 10 questions with a 5-point Likert scale, which is transformed to a score of 1 to 100. To evaluate the prototype software, the PSSUQ was used. It consists of 16 individual questions evaluated on a 7-point Likert scale, in which 1 is defined as “Strongly agree,” and 7 is defined as “Strongly disagree.” The PSSUQ offers three categories: system usefulness, information quality, and interface quality. The SUS and PSSUQ are displayed in Supplementary Appendices 1 and 2 (online only).^8^^,^^9^

#### Number of reattempts

One attempt was defined as the selection of a new position for the first fenestration (starting position). Reattempts were allowed for all observers until they were confident with the configuration. The main goal was to identify the most advantageous position, which was defined as the configuration with minimal overlap with any stent struts. Reattempts were only counted for part II of the experiment, because the phantom stent graft in part I did not feature a stent strut design.

### Statistical analysis

Descriptive statistics were used to report accuracies, time, and usability scores. Means with standard deviation are reported. *t*-tests or Wilcoxon rank-sum tests were performed to compare positional accuracy, time, and usability, depending on the normality of frequency distribution. All the reported *P*-values are descriptive. Statistical analysis was performed using R (R Core Team, The R Foundation).^10^

## Results

### Results of part I of the experiment: Feasibility of fenestration positioning based on an MxR virtual object overlay

#### Positional accuracy

The workflow was successfully completed by all 32 observers (100%). Overall, 128 fenestrations were marked.

The mean distance of the center of the fenestration in the marked stent graft to the ideal position for F1, F2, F3, and F4 was 1.3 mm (standard deviation [SD], 0.6 mm), 1.0 mm (SD, 0.5 mm), 1.0 mm (SD, 0.6 mm), and 1.5 mm (SD, 0.9 mm), respectively.

The mean distances between fenestrations within the marked stent grafts compared with the distances within the ideal stent graft for F2 to F1, F2 to F3, F2 to F4, F1 to F3, F1 to F4, and F3 to F4 were 0.4 mm (SD, 0.5 mm), 0.7 mm (SD, 0.7 mm), 1.0 mm (SD, 0.8 mm), 0.7 mm (SD, 0.7 mm), 1.0 mm (SD, 0.9 mm), and 1.2 mm (SD, 1.0 mm).

**Fig 4:**
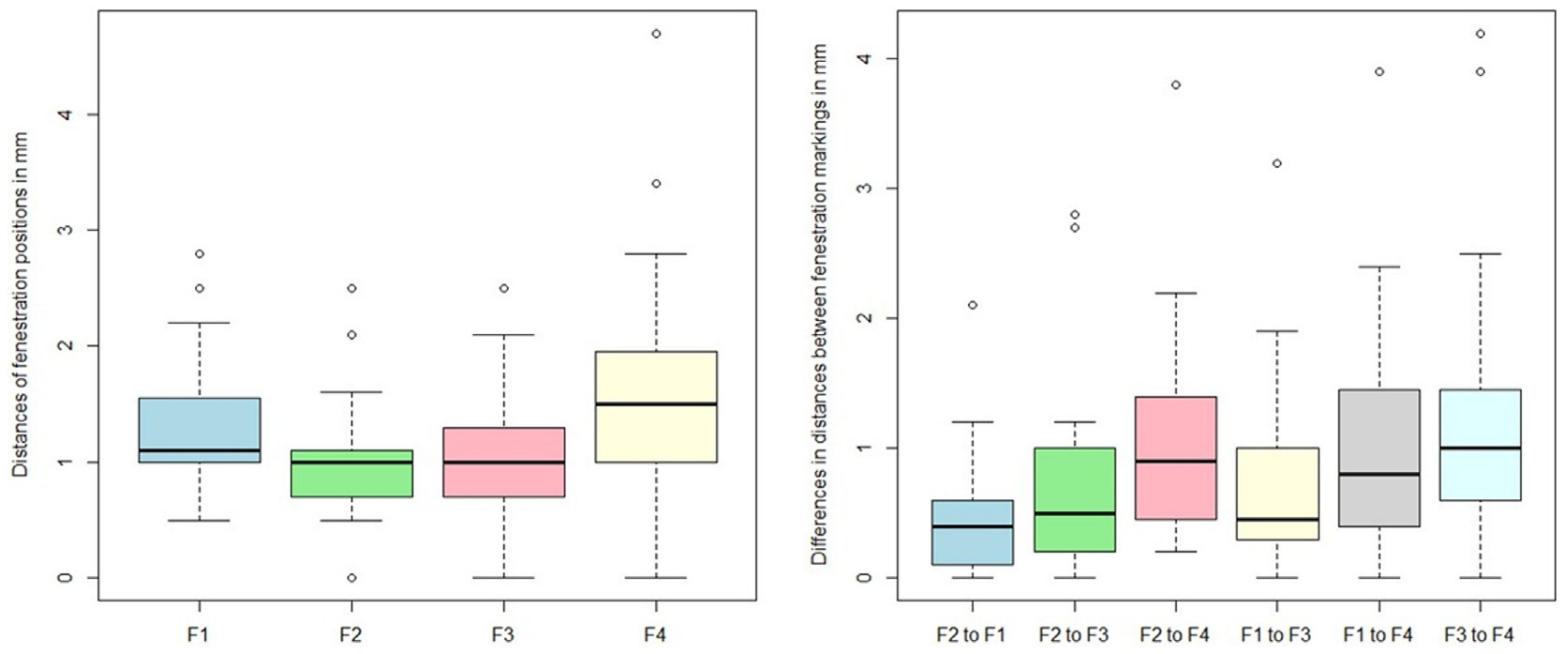
Distances of the center of the fenestration in the marked stent graft to the ideal position for F1 to F4 and differences in distances between fenestrations within the marked stent grafts compared with the distances within the ideal stent graft for F2 to F1, F2 to F3, F2 to F4, F1 to F3, F1 to F4, and F3 to F4.

The mean distance of the centroids of the four fenestrations on the marked stent graft to the centroid of the ideal stent graft was 0.7 mm (SD, 0.4 mm) in the clock position and 0.5 mm (SD 0.4) in cranio-caudal position. Results are presented in Table I and Fig 4.

**Table I:** Distances of the center of the fenestration in the marked stent graft to the ideal position for F1 to F4, difference of distances between fenestrations within the marked stent grafts compared with the distances within the ideal stent graft, and errors of centroid positions

	Mean, mm	SD, mm
Distances of the center of the fenestration in the marked stent graft to the ideal position for F1 to F4		
F1 (TC)	1.3	0.6
F2 (SMA)	1.0	0.5
F3 (RRA)	1.0	0.6
F4 (LRA)	1.5	0.9
Difference of distances between fenestrations within the marked phantom stent grafts compared with the distances within the ideal stent graft		
F2 to F1 (SMA to TC)	0.5	0.5
F2 to F3 (SMA to RRA)	0.7	0.7
F2 to F4 (SMA to LRA)	1.0	0.8
F1 to F3 (TC to RRA)	0.7	0.7
F1 to F4 (TC to LRA)	1.0	0.9
F3 to F4 (RRA to LRA)	1.2	1.0
Distances of centroid positions		
Clock position of centroid	0.7	0.4
Cranio-caudal distance of centroid	0.5	0.4

*LRA,* Left renal artery; *RRA,* right renal artery; *SMA,* superior mesenteric artery; *SD,* standard deviation; *TC,* celiac trunk.

#### Duration

The mean duration of the workflow (steps 2-5) was 5:31 minutes (SD, 1:19 minutes). The fastest observer needed 3:13 minutes, and the slowest observer required 9:02 minutes. Step 2 (Virtual Object Generation) was performed in a mean duration of 2:55 minutes (SD, 0:51 minutes). Marking the stent graft required a mean duration of 2:35 minutes (SD, 0:53 minutes).

#### Usability

The mean score in the SUS evaluating the MxR system’s usability was 84.2 of 100 points (SD, 11.5). The mean score on the PSSUQ evaluating the software components was 1.8 (SD, 0.6) on a scale from 1 to 7. System usefulness was rated with a mean of 1.5 (SD, 0.39), information quality with a mean of 2.2 (SD, 1.1), and interface quality with 1.9 (SD, 0.65).

### Results of part II of the experiment: Comparing the MxR-assisted workflow with a conventional method

#### Positional accuracy

In the second part of the experiment, 48 fenestrations per group (conventional vs MxR) were marked by 12 observers on a phantom stent graft with an incorporated strut design. The mean distance from predefined to marked positions for F1 to F4 were 0.9 mm (SD, 1.2 mm), 0.3 mm (SD, 0.3 mm), 1.2 mm (SD, 0.8 mm), and 0.9 mm (SD, 0.7 mm) for the conventional method, and 1.4 mm (SD, 0.7 mm), 0.6 mm (SD, 0.5 mm), 0.8 mm (SD, 0.7 mm), and 1.2 mm (SD, 0.6 mm) for the MxR-assisted method (*P* = .07).

The mean differences of distances between fenestrations within the marked stent grafts compared with the ideal stent graft for F2 to F1, F2 to F3, F2 to F4, F1 to F3, F1 to F4, and F3 to F4 were 0.4 mm (SD, 0.4 mm), 0.6 mm (SD, 0.7 mm), 0.8 mm (SD, 0.5 mm), 0.7 mm (SD, 0.7 mm), 0.5 mm (SD, 0.5 mm), and 1.1 mm (SD, 0.7 mm) for the conventional group and 0.6 mm (SD, 0.5 mm), 0.5 mm (SD, 0.4 mm), 0.9 mm (SD, 0.6 mm), 0.8 mm (SD, 0.6 mm), 0.9 mm (SD, 0.7 mm), and 1.0 mm (SD, 0.6 mm) for MxR, respectively (*P* = .15).

**Fig 5:**
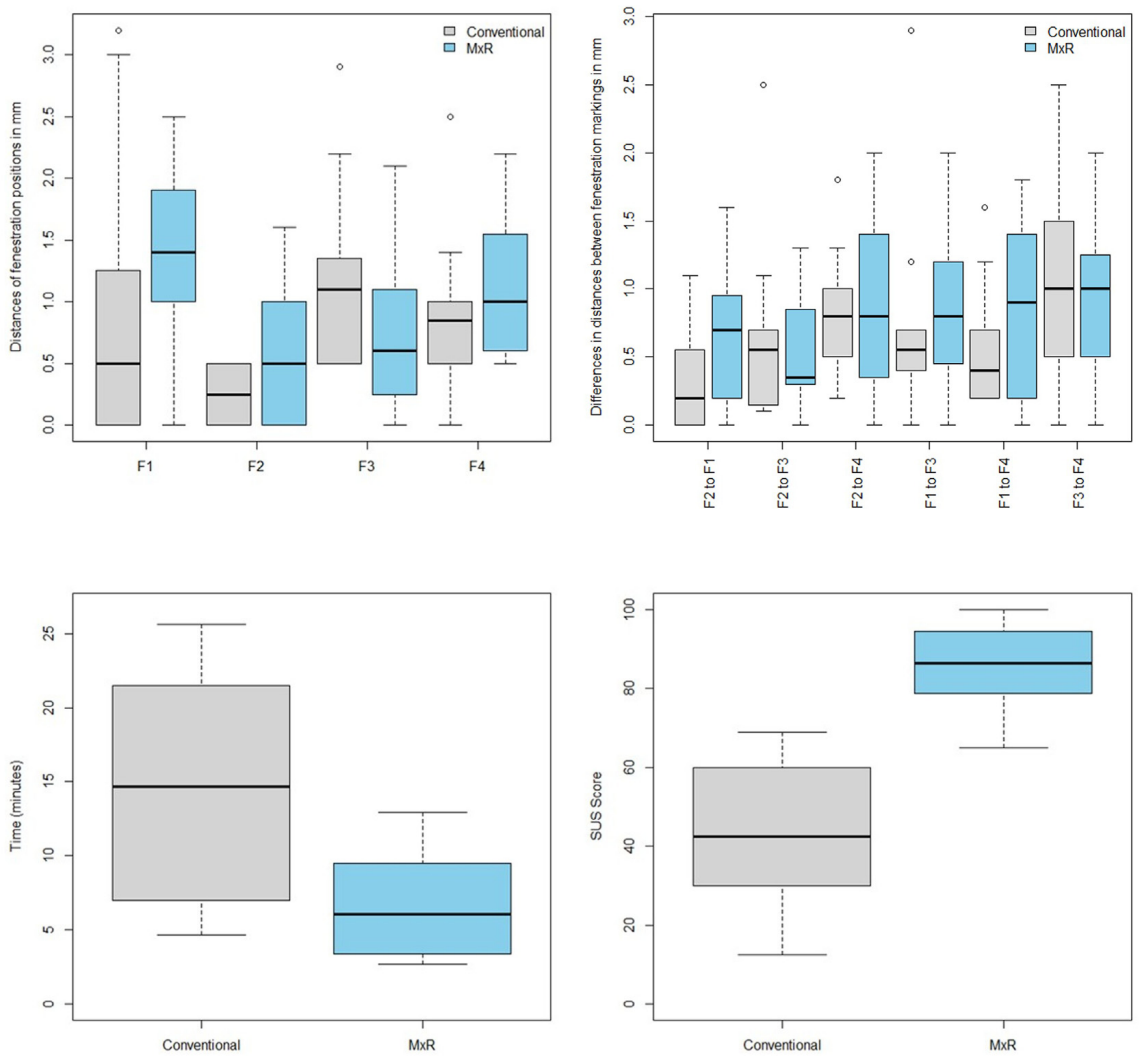
Distances of the center of the fenestration in the marked stent graft to the ideal position for F1 to F4 and differences in distances between fenestrations within the marked stent grafts compared with the distances within the ideal stent graft for F2 to F1, F2 to F3, F2 to F4, F1 to F3, F1 to F4, and F3 to F4, as well as time required and system usability scale scoring, comparing conventional method with the Mixed Reality (MxR)-assisted workflow.

The mean distance of the centroids of the four fenestrations on the marked stent graft to the centroid of the ideal stent graft was 0.4 mm (SD, 0.5 mm) in the clock position and 0.3 mm (SD, 0.3 mm) in the cranio-caudal position for the conventional stent graft, and 0.6 mm (SD, 0.4 mm) and 0.5 mm (SD, 0.3 mm) for MxR, respectively. Results are presented in Table II and Fig 5.

**Table II:** Distances of the center of the fenestration in the marked stent graft to the ideal position for F1 to F4, differences of distances between fenestrations within the marked stent grafts compared with the distances within the ideal stent graft, and errors of centroid positions, comparing the conventional with the Mixed Reality (*MxR*)-assisted workflow

	Conventional, mm		MxR, mm	
	Mean	SD	Mean	SD
Distances of the center of the fenestration in the marked stent graft to the ideal position for F1 to F4				
F1 (TC)	0.9	1.2	1.4	0.7
F2 (SMA)	0.3	0.3	0.6	0.5
F3 (RRA)	1.2	0.8	0.8	0.7
F4 (LRA)	0.9	0.6	1.2	0.6
Differences of distances between fenestrations within the marked phantom stent grafts compared with the distances within the ideal stentgraft				
F2 to F1 (SMA to TC)	0.4	0.4	0.6	0.5
F2 to F3 (SMA to RRA)	0.6	0.7	0.5	0.4
F2 to F4 (SMA to LRA)	0.8	0.5	0.9	0.6
F1 to F3 (TC to RRA)	0.7	0.7	0.8	0.6
F1 to F4 (TC to LRA)	0.5	0.5	0.9	0.7
F3 to F4 (RRA to LRA)	1.1	0.7	1.0	0.6
Distances of centroid positions				
Clock position of centroid	0.4	0.5	0.6	0.4
Cranio-caudal distance of centroid	0.3	0.3	0.5	0.3

*LRA,* Left renal artery; *RRA,* right renal artery; *SD,* standard deviation; *SMA,* superior mesenteric artery; *TC,* celiac trunk.

#### Duration

The conventional method required significantly more time, with a mean of 14.6 minutes (SD, 7.7 minutes) compared with 6.7 minutes (SD, 3.7 minutes) for MxR (*P* < .01).

#### Usability

In the comparative analysis, the mean SUS was 43.2 (SD, 20.0) for the conventional method and 85.1 (SD, 10.3) for MxR (*P* < 0 .01).

#### Number of reattempts

Repeated attempts were needed by 10 of 12 observers in the conventional group compared with three of 12 in the MxR group. Results on duration, usability, and number of reattempts are presented in Table III.

**Table III:** Duration, number of reattempts, and System Usability Score (*SUS*) rating in conventional vs Mixed Reality (*MxR*)-assisted workflows

	Conventional		MxR	
	Mean	SD	Mean	SD
Time, minutes	14.6	7.7	6.7	3.7
Number of reattempts	10/12		3/12	
SUS rating	43.2	20	85.1	10.3

*SD*, Standard deviation.

## Discussion

CMDs are often the preferred treatment option for elective juxtarenal, pararenal, and thoracoabdominal aortic aneurysms.^11^ However, CMDs are associated with delivery times of several weeks and are therefore not necessarily available for emergent endovascular repair. Furthermore, industry-provided CMDs might not be available in cost-sensitive health care systems. In these cases, PMEGs are considered a useful adjunct to the endovascular armamentarium.^1^^,^^2^ There are several options available to determine the position of fenestrations on the physical stent graft when producing PMEGs. The conventional method requires aortic morphometry using standard software and transferring the positions of fenestrations with some form of physical reference according to preprocedural planning.^3^^,^^4^ Another method involves the use of 3D aortic model templates using a 3D printer, with the potential advantage of simulating more closely the actual stent graft position in situ and enhancing accuracy of fenestration locations.^3^^,^^6^ The conventional method lacks standardization, and avoiding stent struts can be challenging and time-consuming by the trial-and-error principle. It might also result in suboptimal compromise. The method with 3D-printed aortic templates takes several hours and requires respective infrastructure for 3D printing and sterilization.^5^^,^^12^, ^13^, ^14^, ^15^ Therefore, there is room for alternative methods.

This study demonstrates the initial feasibility of an MxR-assisted workflow designed to accurately position fenestrations in the context of physician-modified stent grafts. In this experimental setup, the workflow was easy to use and led to precise positioning of planned fenestrations with 1 to 2 mm in positional errors. The very small residual errors for the conventional method likely resulted from measurement inaccuracy due to not perfectly straight or rectangular placement of rulers. In the case of MxR, the small remaining positional errors could have been the result of imperfect registration or slight errors due to perspective on the physical-virtual overlay, which can be difficult to detect. Due to the virtual object approach, the workflow is universally applicable to stent grafts of different manufacturers and sizes and with varying numbers of fenestrations. Furthermore, the materials required were limited. The workflow requires the HMD (Magic Leap 2), as well as the prototype software that can be installed on any personal computer, and the templates that are easily manufactured and that are suited for repeatable use and sterilization. To the best of our knowledge, this is the first prototype using this type of template-MxR-approach for PMEG manufacturing. By combining the virtual object with the physical template and stent graft in our workflow, the unique potential of MxR technology is utilized. Handling and manipulation of the stent graft is minimized. The workflow was quick enough (several minutes) to be used in emergency scenarios, since the design and marking of the stent graft requires only a few minutes. Furthermore, the workflow was assessed by many observers and demonstrated its robustness, requiring very little to no prior experience.

One of the key advantages for future developments is that the virtual object approach could eliminate the manual measurements of lengths and clock positions entirely. As a first step, the present experiment was conducted to investigate the feasibility of marking fenestration positions with a virtual overlay as guidance as well as an initial comparison with a conventional method. Future experiments will focus on the production of ideal stent graft virtual objects without the requirement of human measurements. This could be realized through the export of a volume rendering of a stretched-vessel view that can be scaled to the desired aortic diameter, or through more advanced simulation technology anticipating stent graft positioning within the aorta.^16^^,^^17^ These digital representations of desired stent graft designs will then be used to source the virtual object. Thereby the method can closely mimic the 3D-printed model approach, while eliminating the time constraints and infrastructural requirements associated with printing and sterilization. It is important to note that the patient-individual component of PMEG is represented strictly by virtual objects, rather than physical prints, with all its implications for practical use. Although the materials required are limited and the method does not require any patient-individual 3D-printing and sterilization, it still requires the template as well as the HMD with the associated software (Brainlab AG), all of which are prototypical components that are not yet disseminated throughout vascular surgical departments. Furthermore, similar to 3D-printing, MxR is a rather new technology that most surgeons have no or very limited experience with. Therefore, the method might require some experience to gain sufficient confidence in the final output.

There are several limitations of this study to consider. First, this was a phantom model experiment and did not investigate clinical feasibility. Translation to clinical use needs to be demonstrated. The comparison of the MxR-assisted PMEG workflow with alternative techniques with regard to accuracy, duration, and usability is also subject to limitations. The comparative analysis was limited to one conventional method and one specific stent graft design with relatively narrow struts (32 mm Endurant Cuff). Additionally, the observers in our study were largely not surgeons experienced with complex endovascular repair, which helped demonstrate the robustness of the method, but might have added to an overestimation of the advantages of MxR in time and usability in this experiment. Other methods, including 3D-printed aortic models, were not included and are subject to future studies. Furthermore, the experiments were conducted using a phantom model stent graft, which is partially comparable with a regular stent graft in clinical use. This might have influenced results, especially for the conventional method, because the conventional method was very convenient to use with a perfectly tubular, straight phantom model in a fixed position, compared with actual stent grafts. However, the phantom model approach was chosen to enable exact measurements of positional accuracy, which is more difficult in the case of stent grafts.

## Conclusion

The MxR-assisted workflow enabled accurate, efficient, and user-friendly PMEG planning. At least in this experimental setup, it outperformed the conventional method in usability and speed, supporting its potential for broader clinical applications.

## Funding

This study was supported by the 10.13039/501100017515Heidelberger Stiftung Chirurgie.

## Disclosures

D.B. and C.U. are consulting for Brainlab AG, Munich, Germany. A.M. and N.B. are currently employed at Brainlab AG, Munich, Germany. A patent application related to this work was filed at the European Patent Office in the name of Heidelberg University and RWTH Aachen. Medtronic plc (Galway, Ireland) provided the stent grafts illustrated in the figures free of charge.

## Figures and Tables

**Supplementary Fig 1 (online only):**
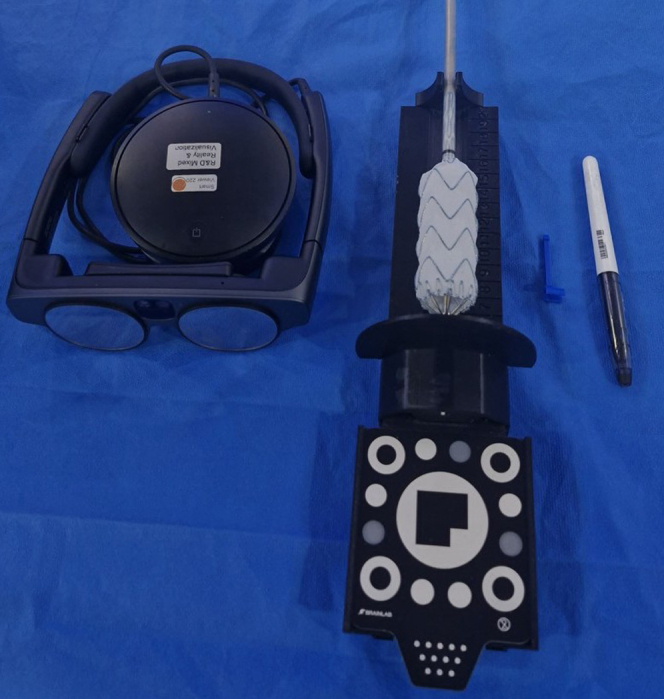


**Supplementary Fig 3 (online only):**