# Supplementary material for: Feasibility of Mixed Reality-assisted physician-modified endografts
Source: J Vasc Surg Cases Innov Tech. 2025 Jun 21;11(5):101889. doi: 10.1016/j.jvscit.2025.101889 (PMC12284275; doi:10.1016/j.jvscit.2025.101889)
Supplement: Supplementary Appendix 1 (online only) [file mmc1.pdf]

System Usability Scale (SUS)

	Strongly Disagree				Strongly Agree
I think that I would like to use this product frequently.	1	2	3	4	5
I found the product unnecessarily complex.	1	2	3	4	5
I thought this product was easy to use.	1	2	3	4	5
I think that I would need the support of a technical person to be able to use this product.	1	2	3	4	5
I found the various functions in this product were well integrated.	1	2	3	4	5
I thought there was too much inconsistency in this product.	1	2	3	4	5
I would imagine that most people would learn to use this product very quickly.	1	2	3	4	5
I found this product very awkward to use.	1	2	3	4	5
I felt very confident using this product.	1	2	3	4	5
I needed to learn a lot of things before I could get going with this product.	1	2	3	4	5
