# Supplementary material for: Feasibility of Mixed Reality-assisted physician-modified endografts
Source: J Vasc Surg Cases Innov Tech. 2025 Jun 21;11(5):101889. doi: 10.1016/j.jvscit.2025.101889 (PMC12284275; doi:10.1016/j.jvscit.2025.101889)
Supplement: Supplementary Appendix 2 (online only) [file mmc2.pdf]

UIUX Trend

PSSUQ Questionnaire PDF Template

On a scale between Strongly Agree to Strongly Disagree, please rate the following statements:

	Strongly Agree					Strongly Disagree		
PSSUQ	1	2	3	4	5	6	7	N.A.
1. Overall, I am satisfied with how easy it is to use this system.								
2. It was simple to use this system.								
3. I was able to complete the tasks and scenarios quickly using this system.								
4. I felt comfortable using this system.								
5. It was easy to learn to use this system.								
6. I believe I could become productive quickly using this system.								
7. The system gave error messages that clearly told me how to fix problems.								
8. Whenever I made a mistake using the system, I could recover easily and quickly.								
9. The information (such as online help, on-screen messages, and other documentation) provided with this system was clear.								

UIUX Trend

	Strongly Agree					Strongly Disagree		
	1	2	3	4	5	6	7	N.A.
10. It was easy to find the information I needed.								
11. The information was effective in helping me complete the tasks and scenarios.								
12. The organization of information on the system screens was clear.								
13. The interface of this system was pleasant.								
14. I liked using the interface of this system.								
15. This system has all the functions and capabilities I expect it to have.								
16. Overall, I am satisfied with this system.								

Questions 1 to 16: Overall
Questions 1 to 6: System Usefulness (SYSUSE)
Questions 7 to 12: Information Quality (INFOQUAL)
Questions 13 to 16: Interface Quality (INTERQUAL)

Source: uiuxtrend.com
